# How the RCOphth CPD system works

**Published:** 2017-05-12

**Authors:** Alex Tytko

**Affiliations:** 1 Head of Education and Training, The Royal College of Ophthalmologists, 18 Stephenson Way, London, UK.

**The RCOphth CPD system is tried and tested and is now on-line and available to members and non-members. Credits are accumulated over a five-year cycle and the record of educational activity is tied to appraisal and revalidation.**

**Figure:**
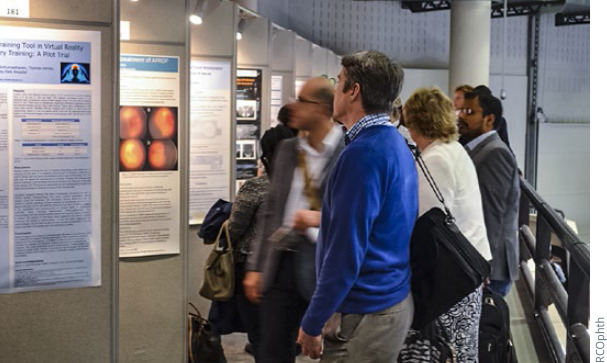
CPD. Viewing posters at the RCOphth Congress. UNITED KINGDOM

The Royal College of Ophthalmologists (RCOphth) started its CPD programme in 1996 and is open to all fellows, members and non-members (non-members are charged an annual fee). The programme is designed for those working in the National Health Service (NHS) as well as those working outside of it. CPD is an online system.

The programme follows a five year cycle of CPD activity in which 250 credits need to be accumulated. Participants are expected to accumulate 50 credits per year. The system allows doctors to record their educational activity which they are then able to present at their appraisal and revalidation. It supports them in specific changes in practice and career development. Points given by the College are based on one point equating to one hour of educational activity.

The College has a mechanism for approving meetings, symposia, conferences etc. for allocation of CPD points. An application is completed and sent to the College for approval. The College holds a list of approved activities that are reviewed every five years in the UK, Ireland and overseas. Any new regular meeting will need to have run successfully for three years or three occasions before being considered for inclusion on the approved list. Meetings accredited by other Colleges for CPD purposes are all recognised by the College.

## CPD categories

Categories assist people to classify CPD and to ensure that a balance of activities is undertaken. There are four categories (see Table 1).

**Table 1:** Examples of educational activities that may qualify for CPD

A. Clinical and Academic: Internal	B. Clinical and Academic: External	C. Clinical and Academic: Self-directed	D. Professional and Managerial
Participating in local teaching programmes	Participating in regional/national and international seminars/workshops	Completing journal self-assessment questions	Attending a course on how to train e.g. training the trainers
Participating in department audit meetings	Attending conferences e.g. Annual Congress	Reviewing a paper for a journal	Attending a meeting on how to use a new piece of software e.g. PowerPoint or other computer related activity
Participating in local seminars and meetings	Making new presentations at conferences	Reading journals and text books or completing an e-learning activity	Attending a course on interview techniques/equality and diversity training etc.
Participating in journal clubs/x-ray/pathology meetings etc.	Undertaking a research project that results in a publication	Undertaking visits to other units	
Participating in online training e.g. webinars, courses		Writing examination questions and examining	

Category A: Clinical and Academic: Internal (10 points)

Category B: Clinical and Academic: External (20 points)

Category C: Clinical and Academic: Self directed (5 points)

Category D: Professional & Managerial (5 points)

## How to record CPD

Participants should record CPD points on a continuous basis and plan what they wish to attend in advance as far as possible. What actually happens is that this is often left to the last minute when preparing for appraisals. All this can be recorded on the online system on a regular basis. CPD records are an excellent way to demonstrate that knowledge and skills are up to date.

It is the responsibility of participants to ensure that they undertake a range of CPDs that reflects the local and national needs of their practice and their own learning needs.

## Keeping a diary

**Figure 1.**
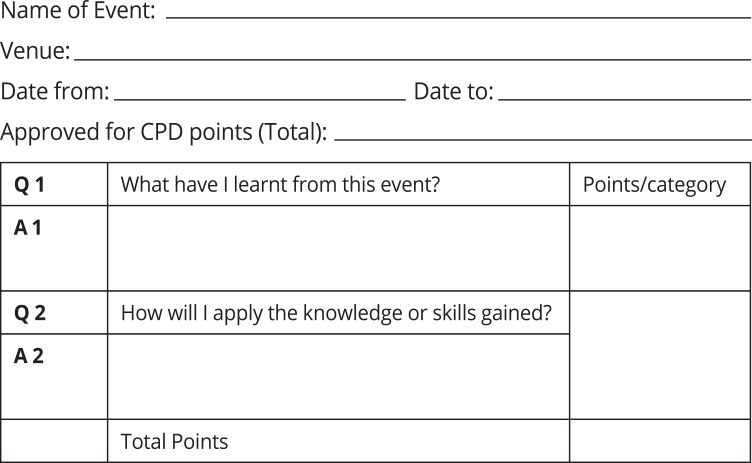
Diary

This does not need to be a complex process and for many years the College used a paper version before it switched to an online system (see Figure 1). This can be as easy as having an ordinary paper diary and writing down your events and points on the days that you attend them. Again this can be prepared in advance and help with the planning of your educational needs. Providing a self-made diary which can be photocopied and made into a booklet and carried round for easy access is also a cost-effective option.

## Reflective practice

Reflection will help a doctor assess whether their learning is adding value to the care of their patients and improving the services in which they work; they should record any impact (or expected future impact) on their performance or practice. Reflection should also occur as soon as possible following the event as this will make it more meaningful.

Reflection drives change in performance and is the key to effective CPD. The General Medical Council's *‘Good medical practice’* (**www.gmc-uk.org/Good_medical_practice___English_1215.pdf_51527435.pdf**) requires you to reflect regularly on your standards of medical practice. Reflection must be integral to your own development plan, appraisal and job planning discussions.

You must reflect on all aspects of your professional work. This should be informed by discussion with others and by specific evidence, such as data from audit, complaints and compliments, significant events, information about service improvements, results of workplace-based assessments and feedback from patients and colleagues.

Where an activity has not been formally approved for CPD, it is the responsibility of the participant to record the activity and document the learning achieved. Learning may reinforce existing good practice as well as provide new knowledge.

How you record your reflective practice is important and examples are given below to demonstrate this.

### Example 1

Individual X attends a full day symposium on glaucoma. The symposium was approved for a total of six CPD points.

A **leading specialist** in the field of glaucoma with a purely glaucoma practice.

**Table:** 

Q	What have I learnt (new knowledge/information) from this event?	Points allocated	Category
**A.**	There was little new at this seminar for me in terms of knowledge. I did hear about a new technique for tying releasable sutures which I will try myself	1	Clinical and Academic External (B)

i.e. only one point out of a possible six allocated as little new knowledge/skills gained.

### Example 2

Individual X attends a full day symposium on glaucoma. The symposium was approved for a total of six CPD points.

A **general ophthalmologist** with no glaucoma subspecialty interest.

**Table:** 

Q	What have I learnt (new knowledge/information) from this event?	Points allocated	Category
**A.**	I learnt all about the new drugs available for the treatment of glaucoma and the mechanisms of action. I also learnt about drugs in first line and second line, and which combinations are most effective.	6	Clinical and Academic External (B)

i.e. sufficient new knowledge/skill gained to allocate the maximum number of points.

### Example 3

An **ophthalmologist** attends a time management course that was approved for two CPD points.

**Table:** 

Q	What have I learnt (new knowledge/information) from this event?	Points allocated	Category
**A.**	I learnt how to prioritise my correspondence, delegate responsibilities and manage my paperwork more efficiently	2	Professional and Managerial (D)

### Example 4

A **consultant ophthalmologist** reads a review article on endophthalmitis following cataract surgery for two hours.

**Table:** 

Q.	What have I learnt (new knowledge/information) from this?	Points allocated	Category
**A.**	Nothing new but my current knowledge was refreshed and consolidated. I will treat these suspected cases with much more urgency.	2	Clinical and Academic: Self Directed (C)
**Reference**	Bacterial Endophthalmitis – Evidence Based Update Ophthalmology 2002; 109: 13-23		
**Critique**	An excellent review article		

*Note: Maximum number of points allocated for reading journals is only five per year, which must be referenced and critiqued*.

